# The SMYD3-MAP3K2 signaling axis promotes tumor aggressiveness and metastasis in prostate cancer

**DOI:** 10.1126/sciadv.adi5921

**Published:** 2023-11-17

**Authors:** Sabeen Ikram, Apurv Rege, Maraki Y. Negesse, Alexandre G. Casanova, Nicolas Reynoird, Erin M. Green

**Affiliations:** ^1^ Department of Biological Sciences, University of Maryland Baltimore County, Baltimore, MD, USA.; ^2^ Grenoble Alpes University, CNRS UMR5309, INSERM U1209, Institute for Advanced Biosciences, Grenoble, France.; ^3^ Marlene and Stewart Greenebaum Comprehensive Cancer Center, University of Maryland School of Medicine, Baltimore, MD, USA.

## Abstract

Aberrant activation of Ras/Raf/mitogen-activated protein kinase (MAPK) signaling is frequently linked to metastatic prostate cancer (PCa); therefore, the characterization of modulators of this pathway is critical for defining therapeutic vulnerabilities for metastatic PCa. The lysine methyltransferase SET and MYND domain 3 (SMYD3) methylates MAPK kinase kinase 2 (MAP3K2) in some cancers, causing enhanced activation of MAPK signaling. In PCa, SMYD3 is frequently overexpressed and associated with disease severity; however, its molecular function in promoting tumorigenesis has not been defined. We demonstrate that SMYD3 critically regulates tumor-associated phenotypes via its methyltransferase activity in PCa cells and mouse xenograft models. SMYD3-dependent methylation of MAP3K2 promotes epithelial-mesenchymal transition associated behaviors by altering the abundance of the intermediate filament vimentin. Furthermore, activation of the SMYD3-MAP3K2 signaling axis supports a positive feedback loop continually promoting high levels of SMYD3. Our data provide insight into signaling pathways involved in metastatic PCa and enhance understanding of mechanistic functions for SMYD3 to reveal potential therapeutic opportunities for PCa.

## Teaser

Regulation of MAPK signaling by protein lysine methylation promotes metastatic properties in prostate cancer.

## INTRODUCTION

Metastatic prostate cancer (PCa) has a 5-year survival rate of less than 30% and is often resistant to treatment (*1*), indicating an urgent need to improve understanding of mechanisms that drive PCa progression and identify clinically actionable vulnerabilities. Aberrant signaling in critical pathways, such as the phosphatidylinositol 3-kinase/Akt/mammalian target of rapamycin (*2*), p53 (*3*), MYC (*4*), and RAS/RAF/mitogen-activated protein kinase (MAPK) (*5*, *6*) signaling pathways, promotes PCa aggressiveness in vitro and metastatic spread in vivo. Protein lysine methylation signaling is also linked to PCa progression. Numerous lysine methyltransferases (KMTs) are overexpressed in primary tumors and metastatic lesions, including NSD2 (*7*), G9a (*8*), and EZH2 (*9*), a known driver of lethal castration-resistant prostate cancer (CRPC) and a key therapeutic target. Misregulation of these enzymes disrupts the chromatin landscape through altered histone methylation patterns and underlies pathological gene expression programs (*10*, *11*). The protein KMT SET and MYND domain 3 (SMYD3) is also highly expressed in PCa (*12*–*14*); however, whether it has a direct molecular role in promoting aggressive phenotypes associated with advanced prostate adenocarcinoma has not been clearly defined.

SMYD3 is predominantly expressed during early embryonic development in mammals, where it regulates myogenesis and differentiation of skeletal and cardiac muscle (*15*–*17*). Its abundance is low in most adult tissues; however, it is frequently overexpressed in solid tumors, including prostate, breast, lung, colorectal, and liver cancers, among others (*13*, *18*–*20*). In these tumor types, SMYD3 overexpression has been implicated in promoting survival, migration, and invasion (*12*, *18*–*20*); therefore, it is critical to clearly identify the molecular oncogenic mechanisms reliant on SMYD3. In lung and pancreatic ductal adenocarcinomas, the major cellular target for SMYD3’s methyltransferase activity is the MAPK, MAPK kinase kinase 2 (MAP3K2) (*18*). Methylation at lysine (K) 260 of MAP3K2 by SMYD3 inhibits binding of the deactivating phosphatase protein phosphatase 2A (PP2A), thereby sustaining aberrant activation of the Ras/Raf/MAPK kinase (MEK)/extracellular-regulated kinase (ERK) signaling axis and promoting tumorigenesis (*18*). In small cell lung cancer, a Ras-independent cancer, SMYD3 methylates another nonhistone substrate, the ubiquitin ligase ring finger protein 113A (RNF113A), promoting resistance to alkylating chemotherapeutics (*21*). Despite the high expression levels of SMYD3 in other tumor types, including PCa, the prosurvival and prometastatic pathways dependent on SMYD3 signaling have not been clearly defined in these cells.

While activating mutations in the Ras/Raf signaling pathways are not common in PCa, approximately 40% of primary tumors and more than 90% of metastatic tumors show expression changes that lead to pathway activation (*2*). In addition, a substantial increase in MAPK signaling is found in tissue samples of advanced-staged tumors with high Gleason scores and in distant metastases (*5*, *6*). Notably, the SMYD3 target MAP3K2 shows a fourfold increase in expression in cancerous prostate compared to benign tissue (*22*). Mouse models combining Ras activation with loss of phosphatase and tensin homolog (Pten), a common genetic lesion in primary prostate tumors, show rapid progression of primary tumors, features of epithelial-mesenchymal transition (EMT), and increased metastatic burden (*6*, *23*). These data suggest that the aberrant activation of the Ras/MAPK pathway may be a critical step in promoting EMT and the development of advanced PCa. These observations have led to efforts to target MAPK signaling to treat metastatic CRPC (mCRPC), particularly using the MEK1/2 inhibitor trametinib (*24*).

In this study, we demonstrate that SMYD3 overexpression promotes PCa cell aggressiveness by enhancing migration, invasion, and anchorage-independent growth and altering adhesive properties of the cells. This role of SMYD3 is dependent on its catalytic activity and, specifically, its methylation of MAP3K2, which maintains constitutive activation of MEK/ERK signaling and alters the abundance of the EMT protein vimentin, contributing to PCa progression. We identify a positive feedback loop in which MEK/ERK activation positively stabilizes protein levels of SMYD3, continually augmenting its high abundance and oncogenic properties. Last, depletion of SMYD3 or inhibition of its catalytic activity substantially improves survival and decreases metastases in mouse orthotopic xenograft models. Together, these data uncover the SMYD3-MAP3K2 signaling axis as a key regulator of PCa aggression and highlight its potential as a therapeutic target for advanced disease.

## RESULTS

### SMYD3 expression increases in high-grade PCa

**Fig. 1.**
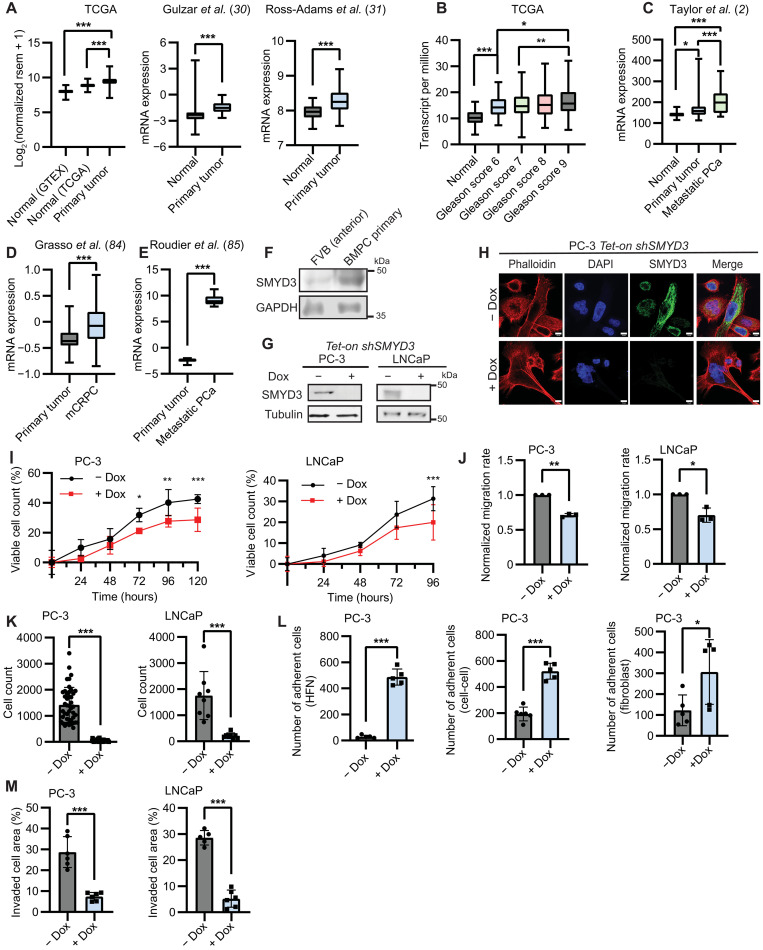
SMYD3 loss impedes PCa progression. (**A**) Expression of *SMYD3* mRNA from TCGA [GTEX normal (*n* = 105), TCGA tumor adjacent normal (*n* = 49), primary tumors (*n* = 495)], Gulzar *et al.* [normal (*n* = 35), primary tumor (*n* = 43)] (*30*), and Ross-Adams *et al.* [normal (*n* = 73), primary tumor (*n* = 126)] (*31*). Significance determined using one-way analysis of variance (ANOVA) and Tukey’s multiple comparisons test for TCGA dataset. (**B**) Expression of *SMYD3* mRNA from TCGA–Prostate Adenocarcinoma in tumor-adjacent normal prostate tissues (*n* = 52), Gleason score 6 (*n* = 45), Gleason score 7 (*n* = 247), Gleason score 8 (*n* = 64), and Gleason score 9 (*n* = 136) prostate tumors. (**C** to **E**) Expression of SMYD3 mRNA from PCa datasets. (C) Taylor *et al.* [normal (*n* = 29), primary tumor (*n* = 131), metastases (*n* = 19)] (*2*). (D) Grasso *et al.* [primary tumor (*n* = 59), metastases (*n* = 32)] (*84*). (E) Roudier *et al.* [primary tumor (*n* = 11), and metastases (*n* = 48)] (*85*). (**F**) SMYD3 immunoblot in primary tumor from BMPC mice (*Hoxb13-MYC^+/−^ Hoxb13-Cre^+/−^ Pten^Fl/Fl^*) (*32*) compared to normal, anterior prostate lobe of FVB mice. (**G**) Immunoblot of PC-3 and LNCaP *Tet-on shSMYD3* cells with and without doxycycline (0.2 μg/ml; −/+ dox) using anti-SMYD3. (**H**) Immunofluorescence of PC-3 *Tet-on shSMYD3* −/+ dox using anti-SMYD3. Scale bars, 10 μm. (**I**) Cell viability time course of PC-3 and LNCaP *Tet-on shSMYD3* cells −/+ dox (*n* = 6). Significance evaluated using two-way ANOVA and Sidak’s multiple comparisons test. (**J**) Normalized migration rate of PC-3 and LNCaP *Tet-on shSMYD3* cells −/+ dox (*n* = 3). (**K**) Soft agar assay of PC-3 (*n* = 42) and LNCaP (*n* = 8) *Tet-on shSMYD3* cells −/+ dox. (**L**) Adhesion of PC-3 *Tet-on shSMYD3* cells −/+ dox to human fibronectin (HFN; *n* = 5) (left), to wild-type PC-3 cells (*n* = 6) (middle), and to NIH3T3 fibroblasts (*n* = 5) (right). (**M**) Invasion capacity of PC-3 and LNCaP *Tet-on shSMYD3* cells (*n* = 3). Significance evaluated using two-tailed unpaired Student’s *t* test. Error bars represent SD, and *P* values are indicated as follows: *P < 0.05, **P < 0.01, and ***P < 0.001.

The *SMYD3* mRNA is highly expressed in multiple tumor types relative to normal tissue and frequently increases further in advanced disease stages (*12*–*14*, *20*, *25*–*28*). Evaluation of *SMYD3* expression in primary prostate tumors from The Cancer Genome Atlas (TCGA) database (*29*) compared to tumor-adjacent (TCGA) or normal prostate tissue [Genotype-Tissue Expression (GTEx)] showed a significant increase in *SMYD3* mRNA levels in tumor samples (Fig. 1A), similar to additional independent datasets of primary prostate tumors [Fig. 1A (*30*, *31*)]. Tumor samples stratified by Gleason score also showed increased *SMYD3* mRNA expression relative to normal tissue, with more *SMYD3* mRNA present in higher-grade tumors (Fig. 1B). Analysis of patient cohorts with mCRPC also showed increased relative expression of *SMYD3* mRNA in metastatic samples compared to primary tumors (Fig. 1, C to E). We also tested SMYD3 abundance in a genetically engineered mouse model that recapitulates the development of human lethal prostate adenocarcinoma and metastasis through *MYC* overexpression and loss of *Pten* in prostatic luminal epithelial cells (*Hoxb13-MYC^+/−^ Hoxb13-Cre^+/−^* *Pten^Fl/Fl^*, or BMPC mice) (*32*). Primary tumors isolated from these mice showed much higher SMYD3 protein abundance compared to tissue from a normal anterior prostate lobe using anti-SMYD3 immunoblotting (Fig. 1F). In total, these data indicate that SMYD3 expression is up-regulated in prostate tumors, particularly at advanced stages, implicating its misregulation in the progression of PCa.

### Loss of SMYD3 abrogates tumor-associated phenotypes of PCa cells in vitro

To determine the role of SMYD3 in oncogenic properties of PCa cells, we generated PC-3 and LNCaP stable cell lines expressing short hairpin SMYD3 (*shSMYD3*) that targets the 3′ untranslated region (3′UTR) of *SMYD3* under control of a doxycycline-inducible promoter (*Tet-on*). PC-3 cells, derived from bone metastases, are androgen independent and have more aggressive properties than androgen-dependent, metastatic lymph node–derived LNCaP cells; therefore, these two cell lines represent different aspects of disease progression (*33*–*35*). We verified knockdown of SMYD3 in these cells using reverse transcription quantitative polymerase chain reaction (RT-qPCR), immunoblotting, and immunofluorescence, which showed no detectable SMYD3 in the presence of doxycycline (Fig. 1, G and H, and fig. S1A). We also generated additional SMYD3 knock-down PC-3 cells using a short hairpin RNA (*shRNA*) targeting a different sequence of the 3′UTR of *SMYD3* [fig. S1B; (*18*)]. Upon SMYD3 knockdown, cell viability of both PC-3 and LNCaP cells was moderately reduced, although the overall rate of proliferation remained similar either with or without SMYD3 (Fig. 1I). However, SMYD3 depletion did substantially impair the rate of collective cell migration in both PC-3 and LNCaP cells (Fig. 1J and fig. S1, C and 1D), and this reduced rate is independent of doxycycline treatment alone (fig. S1E). Given the moderate reduction in proliferation of SMYD3-depleted cells, we also assayed migration upon cell cycle arrest with aphidicolin treatment and observed that SMYD3 knockdown still resulted in decreased migration rate, indicating that SMYD3 driven changes in migration rate were independent of differences in proliferation (fig. S1F).

We also tested the role for SMYD3 in anchorage-independent growth by measuring colony formation in soft agar. We observed no or very little colony formation of either PC-3 or LNCaP cells lacking SMYD3, while control cells grew efficiently in soft agar (Fig. 1K). This disruption to anchorage-independent growth was independent of doxycycline treatment alone (fig. S1G). Spheroids of PC-3 and LNCaP cells with repressed *SMYD3* formed loose aggregates of cells, rather than the compact, three-dimensional structures formed with SMYD3-expressing cells (fig. S1H). To investigate the involvement of SMYD3 in adhesion, we tested PCa cell adhesion to the extracellular matrix component fibronectin, self-adhesion, and adhesion to fibroblasts, which constitute a major component of the prostate tumor microenvironment. In all cases, we observed that loss of SMYD3 substantially increased adhesion to all substrates tested (Fig. 1L and fig. S1, I to K). Last, SMYD3 repression also led to decreased cell invasion capabilities of both PC-3 and LNCaP cells (Fig. 1M and fig. S1, K and L). Together, our findings show that SMYD3 plays a critical role in tumor cell migration, invasion, anchorage-independent growth, as well as cell–extracellular matrix, cell-cell, and cell-fibroblast adhesion in PCa cells in vitro.

### The catalytic activity of SMYD3 is required for its tumorigenic properties in PCa cells

**Fig. 2.**
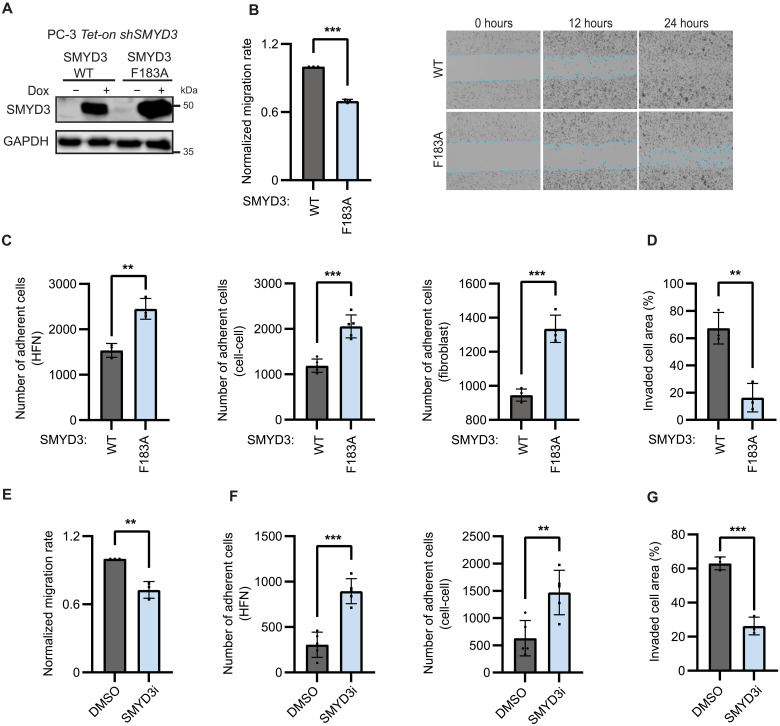
Inhibiting SMYD3 catalytic activity attenuates tumorigenic properties of PCa cells. (**A**) SMYD3 abundance shown by immunoblot in PC-3 *Tet-on shSMYD3* cells reconstituted with either wild-type SMYD3 (*Tet-on SMYD3_WT_*) or SMYD3 with catalytic mutation F183A (*Tet-on SMYD3_F183A_*). (**B**) Normalized migration rate of PC-3 *Tet-on shSMYD3* reconstituted with SMYD3_WT_ and SMYD3_F183A_ (*n* = 3) treated with dox. Representative images of migrating cells are shown at 12 and 24 hours. (**C**) Adhesion of PC-3 *Tet-on shSMYD3* cells treated with dox and reconstituted with SMYD3_WT_ and SMYD3_F183A_ to HFN (*n* = 3) (left), to wild-type PC-3 cells (*n* = 5) (middle), and to NIH3T3 fibroblasts (*n* = 4) (right). (**D**) Invasion capacity of PC-3 *Tet-on shSMYD3* cells treated with dox and reconstituted with SMYD3_WT_ and SMYD3_F183A_. (**E**) Normalized migration rate of PC-3 cells treated with DMSO or SMYD3 inhibitor (SMYD3i; 500 nM EPZ0313686) (*n* = 3). (**F**) Adhesion of PC-3 cells treated with DMSO or SMYD3i to HFN (*n* = 5) (left) and to wild-type, untreated PC-3 cells (*n* = 5) (right). (**G**) Invasion capacity of PC-3 cells treated with DMSO or SMYD3i (*n* = 3). For all panels, error bars represent SD, significance was evaluated using two-tailed unpaired Student’s *t* test, and *P* values are indicated as follows: **P < 0.01 and ***P < 0.001.

Our data show a clear role for SMYD3 in cell migration, adhesion, and invasion of PCa cells; however, it is not known whether these functions rely on the methyltransferase activity of SMYD3 or alternatively, noncatalytic functions of the enzyme. To test this, we generated stable PC-3 and LNCaP cells that ectopically express either wild-type SMYD3 or the catalytically inactive F183A mutant (*18*) at similar levels upon SMYD3 knockdown in the presence of doxycycline (Fig. 2A and fig. S2, A and B). Compared to wild-type SMYD3, expression of SMYD3_F183A_ mutant slowed collective cell migration of PC-3 and LNCaP cells (Fig. 2B and fig. S2C), similar to the phenotype observed with SMYD3 depletion. Cells expressing SMYD3_F183A_ also showed increased adhesion to fibronectin, other PCa cells, and fibroblasts compared to SMYD3_WT_ cells (Fig. 2C and fig. S2D). Last, similar to loss of endogenous SMYD3, SMYD3_F183A_ decreased invasiveness of PCa cells (Fig. 2D).

In addition to assessing phenotypes associated with the SMYD3_F183A_ mutant, we also tested inhibition of SMYD3 catalytic activity using the SMYD3 inhibitor EPZ031686 (SMYD3i), which has been shown to specifically inhibit methyltransferase activity of SMYD3 (*36*). PC-3 cells treated with SMYD3i showed reduced migration rate (Fig. 2E), increased adhesion (Fig. 2F and fig. S2E), and decreased invasiveness (Fig. 2G and fig. S2F) compared to cells treated with the vehicle dimethyl sulfoxide (DMSO). Combined with our findings on the SMYD3_F183A_ mutant, these data demonstrate that the catalytic activity of SMYD3 is required for the tumorigenic behaviors it drives in PCa cells.

### SMYD3 promotes tumor development and metastases of PCa cells in mouse xenograft models

**Fig. 3.**
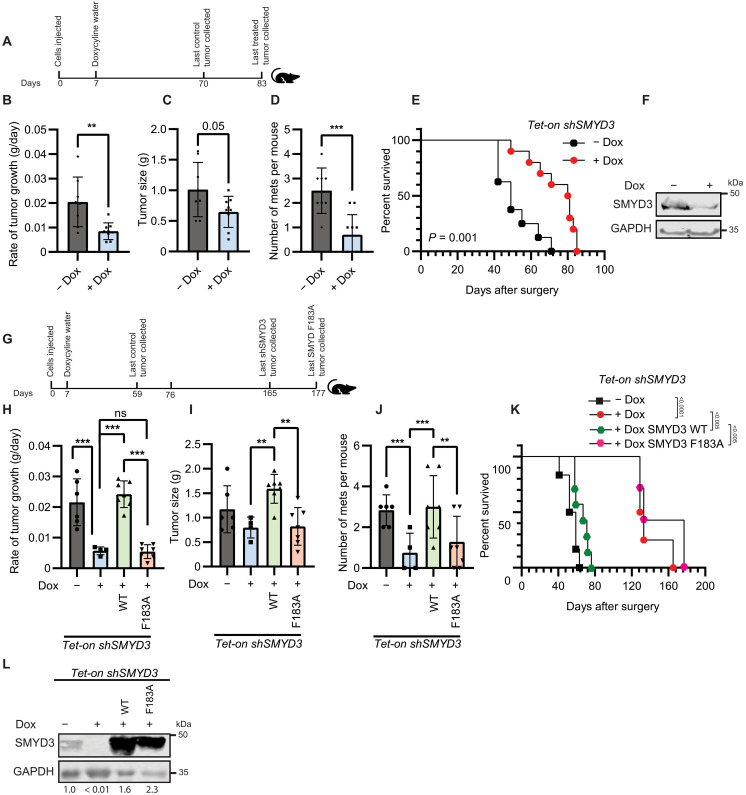
Methyltransferase activity of SMYD3 promotes development of prostate adenocarcinoma and metastases in mouse xenograft models. (**A**) Protocol for PC-3 *Tet-on shSMYD3* orthotopic xenografts in NSG mice −/+ dox (200 μg/ml) treatment in water (*n* = 10 and *n* = 8, respectively). and growth rate of primary tumors (bottom). (**B** to **D**) Rate of tumor growth (B), postcollection primary tumor size (C), and number of metastases per mouse (D) following no treatment or dox treatment in water. For (B) to (D), significance was evaluated using two-tailed unpaired Student’s *t* test. (**E**) Kaplan-Meier survival of NSG mice with PC-3 *Tet-on shSMYD3* orthotopic xenografts −/+ dox treatment in water. Significance was evaluated using log-rank test. (**F**) Immunoblot showing SMYD3 levels in primary prostate tumors dissected from mice −/+ dox treatment. (**G**) Protocol for orthotopic xenografts in NSG mice of PC-3 *Tet-on shSMYD3* cells (−/+ dox, *n* = 6 and *n* = 4, respectively) reconstituted with dox-inducible SMYD3_WT_ (+dox, *n* = 7) or SMYD3_F183A_ (+dox, *n* = 7). (**H** to **J**) Growth rate of primary tumors (H), postcollection primary tumor size (I), and number of metastases per mouse (J) following no treatment or dox treatment in water. For (H) to (J), significance was evaluated using one-way ANOVA and Tukey’s multiple comparisons test. (**K**) Kaplan-Meier survival of NSG mice with PC-3 *Tet-on shSMYD3* orthotopic xenografts reconstituted with dox-inducible SMYD3_WT_ or SMYD3_F183A_ −/+ dox treatment in water. Significance was evaluated using log-rank test. (**L**) Immunoblot showing SMYD3 levels in primary prostate tumors dissected from mice −/+ dox treatment. Values shown under the blot indicate SMYD3 levels relative to glyceraldehyde-3-phosphate dehydrogenase (GAPDH). For all panels, error bars represent SD, and *P* values are indicated as follows: **P < 0.01 and ***P < 0.001; ns, not significant.

We next investigated the role of SMYD3 on tumor behavior in vivo by generating flank and orthotopic mouse xenografts of PC-3 *Tet-on shSMYD3* cells. First, these cells were injected subcutaneously into the hind flank of immune-compromised NSG (NOD.Cg*-Prkdc^scid^ Il2rg^tm1Wjl^/SzJ*) mice and doxycycline was administered in water to repress SMYD3 expression. A statistically significant decrease in tumor volume was observed upon SMYD3 repression compared to the control cells (fig. S3, A and B), suggesting a role for SMYD3 in tumor growth in vivo. To better understand the effect of SMYD3 on primary tumor growth within the prostate microenvironment and evaluate its potential role in metastasis, we next generated orthotopic xenografts by surgically implanting PC-3 *Tet-on shSMYD3* cells into the anterior prostate lobe of NSG mice and administering doxycycline-treated water (Fig. 3A). Depletion of SMYD3 in the xenografts led to a substantially slower tumor growth rate (Fig. 3B) and a reduction in final tumor weight (Fig. 3C and fig. S3C). Notably, there were also fewer metastatic sites upon loss of SMYD3 in the xenografts compared to control mice (Fig. 3D and fig. S3D). Consistent with this observation, SMYD3 knockdown also significantly enhanced survival of the mice (Fig. 3E). Knockdown of SMYD3 in the tumors was confirmed using immunoblotting (Fig. 3F).

We next assessed whether the decreased tumor growth and metastatic burden caused by repression of SMYD3 in the PC-3 xenografts could be rescued by expressing wild-type SMYD3 or the catalytically inactive F183A mutant (Fig. 3G). As previously observed, knockdown of SMYD3 in the xenografts resulted in significantly reduced tumor growth rate compared to untreated controls (Fig. 3H). Ectopic expression of SMYD3_WT_ restored the growth rate, whereas SMYD3_F183A_ expression caused a similarly slow rate of tumor growth as cells without SMYD3 (Fig. 3H). There was also restoration of tumor volume in SMYD3_WT_ xenografts and not with the SMYD3_F183A_ mutant (Fig. 3I and fig. S3E). In addition, mice with either SMYD3 knockdown alone or with ectopic expression of SMYD3_F183A_ showed fewer metastatic lesions compared to mice carrying SMYD3_WT_ xenografts (Fig. 3J). Last, restored expression of SMYD3_WT_, but not the SMYD3_F183A_ mutant, also decreased the survival of mice that which had endogenous SMYD3 repressed in the xenografts (Fig. 3K). Knockdown of SMYD3 and expression of SMYD3_WT_ or SMYD3_F183A_ was confirmed in tumor samples using immunoblotting (Fig. 3L). Together, these data suggest that the catalytic activity of SMYD3 supports tumor growth and promotes metastatic spread in vivo.

### Methylation of MAP3K2 by SMYD3 promotes aggressive properties of PCa cells

**Fig. 4.**
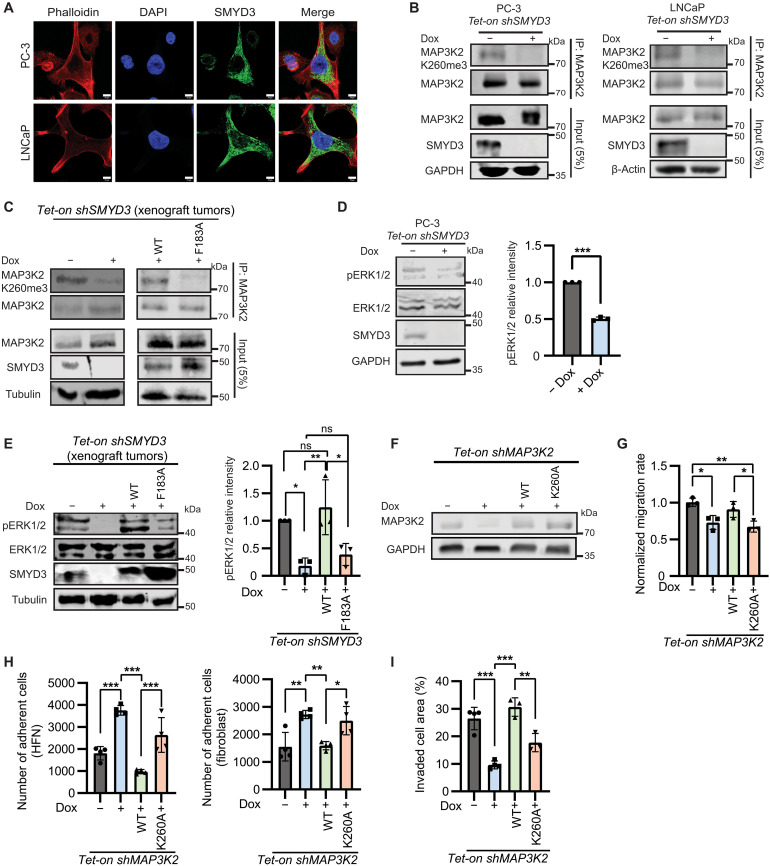
SMYD3 methylation at K260 of MAP3K2 drives tumorigenicity in PCa. (**A**) Immunofluorescent images of PC-3 and LNCaP cells stained with SMYD3 antibody (green), 4′,6-diamidino-2-phenylindole (DAPI) (blue), and phalloidin (red). Scale bars, 10 μm. (**B**) Immunoprecipitation (IP) of endogenous MAP3K2 from PC-3 and LNCaP *Tet-on shSMYD3* cells −/+ dox treatment. IP immunoblots probed with anti-MAP3K2 K260me3 and anti-MAP3K2 antibodies. (**C**) Immunoblots of MAP3K2 IP from PC-3 *Tet-on shSMYD3* orthotopic xenograft tumors reconstituted with SMYD3_WT_ or SMYD3_F183A_, as in Fig. 3 (G to L). IP immunoblots probed with anti-MAP3K2 K260me3 and anti-MAP3K2. (**D**) Immunoblots using anti–phospho-ERK1/2, anti-ERK1/2, and anti-SMYD3 in PC-3 *Tet-on shSMYD3* cells −/+ dox treatment with intensity of phospho-ERK1/2 signal relative to total ERK1/2 normalized to GAPDH plotted on the right (*n* = 3). (**E**) Immunoblots of phospho-ERK1/2 and total ERK1/2 from orthotopic xenograft tumors as described for Fig. 3C. Intensity of phospho-ERK1/2 signal relative to total ERK1/2 normalized to tubulin plotted on the right (*n* = 3). (**F**) MAP3K2 abundance shown by immunoblot in PC-3 *Tet-on shMAP3K2* cells reconstituted with either wild-type MAP3K2 (*Tet-on MAP3K2_WT_*) or MAP3K2 with methyl-inhibiting mutation K260A (*Tet-on MAP3K2_K260A_*). (**G**) Normalized migration rate of PC-3 *Tet-on shMAP3K2* −/+ dox treatment and reconstituted with MAP3K2_WT_ and MAP3K2_K260A_ (*n* = 3) treated with dox. (**H**) Adhesion of PC-3 *Tet-on shMAP3K2* cells −/+ dox treatment and reconstituted with MAP3K2_WT_ and MAP3K2_K260A_ to HFN (*n* = 4) (left) and to NIH3T3 fibroblasts (*n* = 4) (right). (**I**) Invasion capacity of PC-3 *Tet-on shMAP3K2* cells −/+ dox treatment and reconstituted with MAP3K2_WT_ and MAP3K2_K260A_. For all panels, error bars represent SD, significance was evaluated using one-way ANOVA and Tukey’s multiple comparisons test, and *P* values are indicated as follows: *P < 0.05, **P < 0.01, and ***P < 0.001.

Our results have demonstrated a role for the catalytic methyltransferase activity of SMYD3 in phenotypes linked to tumor development and progression of PCa. However, the specific substrate targeted by SMYD3 to drive tumorigenic phenotypes in PCa cells is not known. SMYD3 has multiple biochemically and physiologically validated substrates, including histone H4 (*37*) and RNF113A in the nucleus (*21*) and MAP3K2 in the cytoplasm (*18*). Depending on cell type, SMYD3 has been reported to be present in both the cytoplasm (*18*, *38*, *39*) and the nucleus (*20*), though often predominantly cytoplasmic (*40*). We assayed subcellular distribution of SMYD3 in the PCa cell lines PC-3 and LNCaP and found it primarily in the cytoplasm with minimal nuclear localization (Fig. 4A and fig. S4A). This suggested that SMYD3’s predominant substrate in PCa cells is more likely to be the cytoplasmic protein MAP3K2, rather than nuclear proteins. To determine whether MAP3K2 is methylated in PCa cells, we used an antibody recognizing SMYD3-dependent MAP3K2 K260me3 (*18*). In PC-3 and LNCaP cells, methylation at K260 was detectable on endogenous MAP3K2 in the presence of SMYD3 but lost upon its depletion (Fig. 4B and fig. S4, B and C), indicating that SMYD3 is actively methylating MAP3K2 in these cells. We confirmed SMYD3-dependent MAP3K2 K260 methylation in the orthotopic xenograft tumors of PC-3 *Tet-on shSMYD3* cells (Fig. 4C). Furthermore, reconstitution with SMYD3_WT_ restored MAP3K2 methylation, whereas the methyl mark was lost upon expression of catalytically inactive SMYD3_F183A_ in the tumors (Fig. 4C). As demonstrated in other cell types (*18*), we also tested whether SMYD3 promoted constitutive activation of MEK/ERK signaling in PCa cells. Loss of SMYD3 decreased levels of phosphorylated ERK1/2 in PC-3 and LNCaP cells (Fig. 4D and fig. S4D). Consistently, phosphorylated ERK1/2 was reduced upon loss or inactivation of SMYD3 in the orthotopic xenograft tumors (Fig. 4E), suggesting that SMYD3-catalyzed methylation of MAP3K2 promotes persistent activation of the MEK/ERK pathway in PCa cell lines and tumors.

To assess whether methylation of MAP3K2 at K260 drives aggressive properties of PCa cells linked to high levels of SMYD3 expression, we tested cancer cell phenotypes in PC-3 *Tet-on shMAP3K2* cells and cells in which the knockdown is complemented with either wild-type or K260A mutant MAP3K2, which cannot be methylated (Fig. 4F). In cell migration assays, depletion of MAP3K2 reduced the rate of migration, which was restored with expression of MAP3K2_WT_ but not MAP3K2_K260A_ (Fig. 4G). These data suggest that both loss of function of MAP3K2 and blocking methylation by SMYD3 at K260 phenocopy the outcome of depleting SMYD3 on collective cell migration. Likewise, adhesion of PCa cells to matrix components and fibroblasts was increased (Fig. 4H), and invasion decreased (Fig. 4I) in cells with MAP3K2 knocked down or expressing MAP3K2_K260A_ compared to cells with wild-type MAP3K2. We also tested whether inhibition of the pathway downstream of MAP3K2 similarly altered the properties of PCa cells using BVD-523 (ulixertinib) (*41*), a catalytic ERK1/2 inhibitor (ERKi). PC-3 and LNCaP cells treated with ERKi showed decreased migration, increased adhesion, and lowered invasion capacity compared to DMSO-treated controls (fig. S4, E to G). Together, these data suggest that loss of MAP3K2 activity through depletion of the full-length protein or inhibiting its methylation at K260 phenocopies disruption of SMYD3’s catalytic activity, which is likely driven by overactivation of MAP3K2 and increased ERK1/2 activation to promote aggressive properties of PCa cells.

### Signaling via SMYD3 and MAP3K2 methylation promotes EMT properties of PCa cells by regulating the abundance of vimentin

**Fig. 5.**
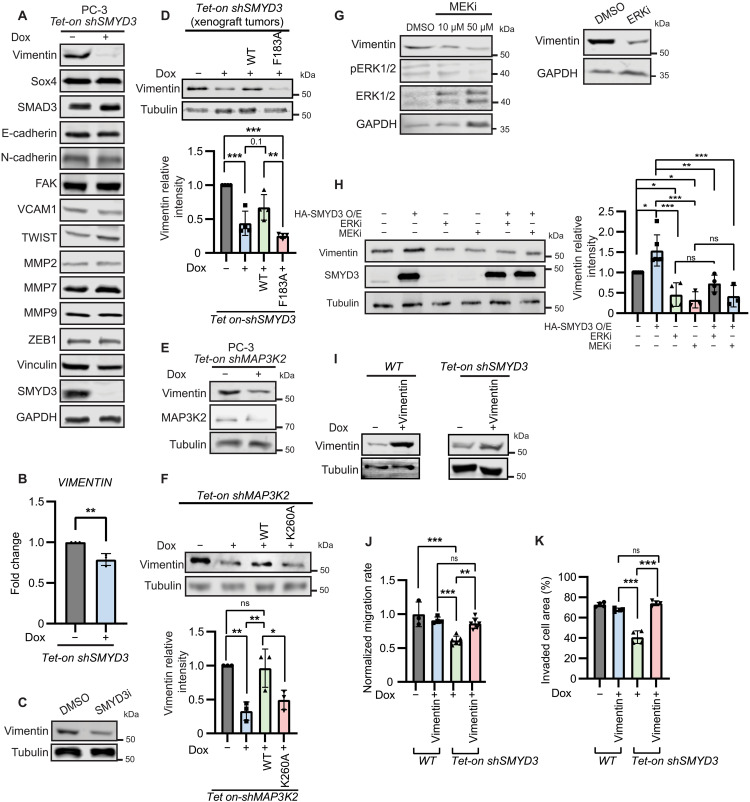
SMYD3 regulates the abundance of vimentin via MAPK signaling in PCa. (**A**) Immunoblots of EMT-associated proteins from PC-3 *Tet-on shSMYD3* cells −/+ dox. (**B**) Fold change of *vimentin* mRNA in PC-3 *Tet-on shSMYD3* cells −/+ dox measured by RT-qPCR. (**C**) Immunoblot of vimentin in PC-3 cells treated with DMSO or SMYD3i (500 nM). (**D**) Immunoblots of vimentin from orthotopic xenograft tumors as described for Fig. 3C. Intensity of vimentin signal relative to tubulin and normalized to wild-type (WT) plotted below (*n* = 4). (**E**) Immunoblot of vimentin from PC-3 *Tet-on shMAP3K2* cells −/+ dox. (**F**) Immunoblot of vimentin in PC-3 *Tet-on shMAP3K2* expressing MAP3K2_WT_ or MAP3K2_K260A_ and intensity of vimentin signal relative to tubulin and normalized to WT is plotted below (*n* = 4). (**G**) Immunoblots of vimentin and phospho-ERK1/2 (pERK1/2) from PC-3 cells with 10 and 50 μM MEKi (left) and from PC-3 cells with 4 μM ERKi (right). (**H**) Immunoblot of vimentin from PC-3 cells with overexpressed *HA-SMYD3* −/+ ERKi (4 μM) or MEKi (50 μM) treatment. Intensity of vimentin relative to tubulin and normalized to WT PC-3 plotted on the right. (**I**) Immunoblot of vimentin in PC-3 *WT* and PC-3 *Tet-on shSMYD3* cells each with *Tet-on VIMENTIN* −/+ dox treatment. (**J**) Normalized migration rate of PC-3 *WT* (*n* = 3) and PC-3 *Tet-on shSMYD3* (*n* = 4) cells each with *Tet-on VIMENTIN* (*n* = 6 for each cell type) −/+ dox. (**K**) Invasion capacity of PC-3 *WT* and PC-3 *Tet-on shSMYD3* [each with *Tet-on VIMENTIN* (*n* = 4 for each cell type) −/+ dox]. For all panels, error bars represent SD, significance was evaluated using an unpaired Student’s *t* test (B) or one-way ANOVA and Tukey’s multiple comparisons test [(D), (F), (H), (J), and (K)], and *P* values are indicated as follows: *P < 0.05, **P < 0.01, and ***P < 0.001.

A major consequence of constant activation of Ras/Raf/MEK/ERK signaling is the up-regulation or activation of factors that drive EMT, promoting tumor progression and metastasis in many cancer types (*6*, *42*, *43*). To evaluate the role of SMYD3-dependent methylation of MAP3K2 during EMT in PCa cells, we monitored the relative abundance of a series of well-validated markers that define epithelial or mesenchymal states (*42*, *44*–*59*) in PC-3 cells and LNCaP cells with depleted SMYD3. The most substantial change we observed in the absence of SMYD3 was a notable reduction in the amount of vimentin (Fig. 5A and fig. S5, A and B), a mesenchymal marker linked to tumor aggressiveness (*57*, *60*, *61*). The transcript encoding vimentin was also modestly reduced upon SMYD3 knockdown (Fig. 5B and fig. S5C), suggesting that SMYD3 may regulate vimentin mRNA transcription or stability, in addition to likely influencing its protein synthesis or degradation. SMYD3 catalytic activity is required for maintenance of vimentin levels, as treatment of PC-3 cells with the catalytic inhibitor EPZ031686 (SMYD3i) decreased vimentin abundance (Fig. 5C). Moreover, we observed reduced abundance of vimentin in the orthotopic xenografts lacking SMYD3 or its catalytic activity (Fig. 5D), suggesting a role for SMYD3 in controlling vimentin levels in vivo. Vimentin levels were also reduced upon loss of MAP3K2 (Fig. 5E), which could be rescued by expression of wild-type MAP3K2 but not the K260A mutant (Fig. 5F). Direct inhibition of MEK using trametinib (MEKi) (*62*) and ERK1/2 inhibition also both reduced vimentin levels in PCa cells (Fig. 5G and fig. S5D).

Together, these data suggest a model in which SMYD3, MAP3K2 activity, and the MEK/ERK cascade all support high abundance of vimentin in PCa cells. To directly test whether the role for SMYD3 in controlling vimentin relies on downstream MAPK signaling, we used cells overexpressing SMYD3, which have increased vimentin abundance, and inhibited the pathway downstream of SMYD3 using either MEKi or ERKi (Fig. 5H). SMYD3 overexpression did not lead to increased vimentin when either MEK or ERK1/2 was inhibited. This suggests that SMYD3 depends on the activity of these kinases to control vimentin abundance and indicates SMYD3 functions upstream of these kinases in regulating vimentin. To further test whether the reduction in vimentin levels in SMYD3-depleted cells was responsible for altering their EMT-associated phenotypes, we ectopically expressed vimentin in PC-3 and LNCaP cells (Fig. 5I and fig. S5D). In SMYD3 knockdown cells expressing ectopic vimentin to restore its levels, we found that the cell migration rate (Fig. 5J and fig. S5F) and invasion capacity (Fig. 5K and fig. S5G) approached that observed for wild-type cells with endogenous SMYD3 expression. Ectopic expression of vimentin in wild-type PC-3 and LNCaP cells did not markedly alter the migration rate (Fig. 5J and fig. S5F) or invasiveness (Fig. 5K and fig. S5G) of these cells, indicating that increased vimentin does not inherently increase migration or invasion in PCa cells. These data demonstrate that SMYD3-dependent control of vimentin abundance contributes to EMT and the aggressive properties of PCa cells.

### A positive feedback loop regulates SMYD3 expression via MEK/ERK signaling

**Fig. 6.**
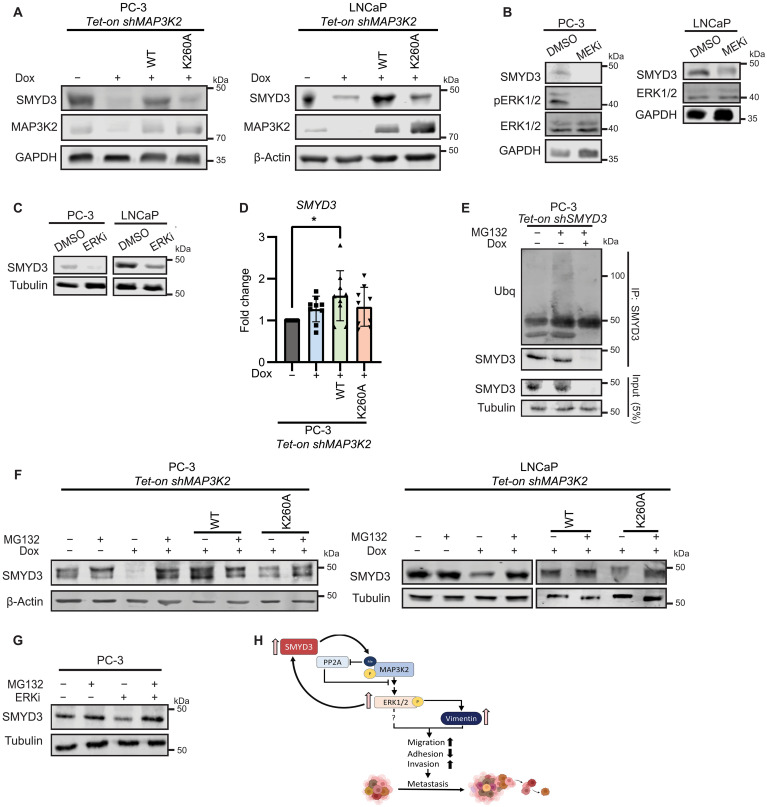
A positive feedback loop regulates SMYD3 abundance in PCa cells. (**A**) Immunoblots of SMYD3 and MAP3K2 levels from PC-3 and LNCaP *Tet-on shMAP3K2* cells −/+ dox treatment reconstituted with MAP3K2_WT_ and MAP3K2_K260A_. (**B**) Immunoblots of SMYD3, pERK1/2, and ERK1/2 from PC-3 and LNCaP cells treated with DMSO or 30 μM MEKi. (**C**) Immunoblots of SMYD3 from PC-3 and LNCaP cells following treatment with DMSO or 4 μM ERKi. (**D**) Fold change of *SMYD3* mRNA abundance in PC-3 *Tet-on shMAP3K2* cells −/+ dox treatment and reconstituted with MAP3K2_WT_ and MAP3K2_K260A_ measured by RT-qPCR. (**E**) Immunoblots of SMYD3 IPs probed with anti-ubiquitin (Ub) and anti-SMYD3 from PC-3 *Tet-on shSMYD3* cells −/+ dox treatment and following MG-132 treatment (22 μM for 7 hours). (**F**) SMYD3 levels from PC-3 and LNCaP *Tet-on shMAP3K2* cells −/+ dox treatment reconstituted with MAP3K2_WT_ and MAP3K2_K260A_ and following MG-132 treatment. (**G**) Immunoblots of SMYD3 from PC-3 cells treated with DMSO or 4 μM ERKi and following MG-132 treatment. (**H**) Model summarizing the SMYD3-MAP3K2 axis and its feedback regulation in PCa. SMYD3 methylates MAP3K2 in PCa cells, which is known to block PP2A binding and promote its constitutive activation and the activation of downstream kinases including MEK1/2 and ERK1/2. This alters migratory, adhesive, and invasive properties of PCa cells to support metastatic spread, at least partly through the regulation of vimentin, and likely additional factors. Methylation of MAP3K2 and activation of MEK/ERK signaling also supports increased abundance of SMYD3 via a posttranslational mechanism limiting proteasomal-dependent degradation of SMYD3.

While evaluating phenotypes in PCa cells with MAP3K2 knockdown, we noted a consistent reduction in the abundance of SMYD3 in both PC-3 and LNCaP cells (Fig. 6A). We also observed that SMYD3 levels were restored upon reconstitution with MAP3K2_WT_ but not with the MAP3K2_K260A_ mutant (Fig. 6A), suggesting that methylation by SMYD3 may regulate its own abundance. We next assessed whether the altered SMYD3 protein abundance was a consequence of disrupted MEK/ERK signaling downstream of MAP3K2 by monitoring SMYD3 levels following inhibition of this pathway with MEKi and ERKi. Similar to knockdown of MAP3K2, both kinase inhibitors caused a clear reduction in SMYD3 abundance in both PC-3 and LNCaP cell lines (Fig. 6, B and C).

To determine whether the amount of SMYD3 is controlled through transcriptional or posttranscriptional mechanisms, we assessed SMYD3 mRNA and protein levels in the cells lacking MAP3K2 or carrying the nonmethylatable MAP3K2_K260A_ mutant. There was little change in *SMYD3* mRNA upon depletion of MAP3K2, and *SMYD3* mRNA was slightly increased with ectopic expression of either MAP3K2_WT_ or MAP3K2_K260A_ (Fig. 6D), suggesting that neither MAP3K2 nor its methylation at K260 is directly influencing the transcription of *SMYD3*. We therefore evaluated the role for proteasomal-mediated regulation of SMYD3 abundance. First, endogenous SMYD3 immunoprecipitated from PC-3 cells was ubiquitinated in the presence of the proteasome inhibitor MG-132, as indicated by recognition of SMYD3 and higher–molecular weight species with an antiubiquitin antibody (Fig. 6E). Furthermore, SMYD3 levels were stabilized in the presence of MG-132 in both MAP3K2-depleted cells and those expressing MAP3K2_K260A_ in both the PC-3 and LNCaP cell lines (Fig. 6F). In addition, the decreased abundance of SMYD3 observed in the presence of ERKi was also stabilized following MG-132 treatment (Fig. 6G). These data indicate that SMYD3 is subject to posttranslational regulation and that lowered activity of MAP3K2, and subsequently ERK1/2, enhances the proteasomal-dependent degradation of SMYD3. Together, these data suggest that SMYD3-dependent signaling to MAPKs drives a positive feedback loop maintaining high abundance of SMYD3 in PCa cells, likely regulated by a target downstream of active ERK1/2 that controls SMYD3 protein abundance (Fig. 6H).

## DISCUSSION

The protein methyltransferase SMYD3 is frequently overexpressed in solid tumors and is often linked to disease progression and aggressiveness (*18*–*21*). Because of its low abundance in adult somatic tissues (*63*, *64*), SMYD3 represents a potential therapeutic target in multiple cancer types. However, the molecular pathway through which it drives cancer progression has not been fully defined in many of the cancers with *SMYD3* overexpression. Our study demonstrates that high levels of SMYD3 in PCa are associated with more aggressive phenotypes linked to EMT-related properties in vitro and increased metastatic spread in vivo. This function for SMYD3 depends on its catalytic activity and appears to be largely mediated through the methylation of its substrate MAP3K2, as shown both in vitro and in xenograft tumors in vivo. Depletion of SMYD3 or mutation of its catalytic site causes phenotypes in PCa cells similar to disrupting its constitutive activation by K260 methylation or inhibiting the downstream MEK and ERK1/2 kinases. Given the predominantly cytoplasmic localization of SMYD3 in PCa, these data support a model in which SMYD3 methylation of a nonhistone substrate promotes aggressiveness and EMT-associated behaviors in PCa cells. While some studies have linked SMYD3 overexpression in cancer cells to transcriptional regulation via its activity on histone 3 lysine 4 (H3K4) methylation, biochemical evidence indicates that H3K4 is not a direct target for methylation by SMYD3 (*37*) and SMYD3 is predominantly cytoplasmic in many cell types (*40*). Our data further support previous findings (*18*, *21*) that there are multiple mechanisms through which signaling pathways and transcriptional outputs may be altered downstream of SMYD3, highlighting the importance of evaluating the role for mechanisms outside of direct chromatin regulation by SMYD3 in tumor initiation and progression.

The activation of Ras/MAPK signaling is frequently observed in advanced and metastatic PCa, promoting transcriptional programs linked to EMT (*6*, *23*). The type III intermediate filament vimentin is a signature of EMT and is frequently up-regulated during tumor progression (*61*, *65*). Notably, vimentin is overexpressed in poorly differentiated and highly metastatic PCa, and high vimentin expression is associated with the acquisition of androgen independence and shorter-duration disease-free survival (*57*, *60*, *61*, *66*). Vimentin is frequently reported as a downstream target of MEK/ERK signaling through regulation of its transcription or protein abundance (*67*–*70*). MAP3K2 has also specifically been demonstrated to regulate the abundance of vimentin in some cancers, such as in triple-negative breast cancer (*68*). In addition, in multiple solid tumor types, SMYD3 has been reported to promote EMT largely through regulation of expression of EMT genes, including *vimentin* (*20*, *27*, *71*). Of the EMT proteins we evaluated in SMYD3-depleted PCa cells, only vimentin showed a substantial change in protein abundance. Restoring vimentin expression in the absence of SMYD3 increased the migration rate and invasion capacity of these cells, suggesting that regulation of vimentin is a key downstream target of SMYD3 signaling driving EMT characteristics of PCa cells. On the basis of the relatively modest change in *vimentin* mRNA abundance in SMYD3-depleted cells, SMYD3-dependent regulation of vimentin levels likely incorporates some transcriptional control as well as modulation of vimentin protein synthesis, degradation, or stability. This is consistent with multiple mechanisms contributing to vimentin abundance through MEK/ERK signaling (*67*–*70*).

Our findings also revealed a positive feedback loop driving SMYD3 expression in PCa cells through MAP3K2-dependent regulation of SMYD3 protein abundance. We observed that depletion of MAP3K2, mutation of the K260 methylation site, or inhibition of the signaling pathway downstream of MAP3K2 all lowered the abundance of SMYD3, indicating that disrupting the SMYD3-MAP3K2 signaling pathway at multiple points altered the feedback regulation of SMYD3. While there has been some investigation into mechanisms controlling *SMYD3* mRNA expression, such as by the transcription factor E2F1 (*25*, *72*, *73*) and others, there are limited data regarding regulation of SMYD3 protein abundance through other mechanisms. Our data show that poly-ubiquitinated SMYD3 is detectable upon proteasome inhibition and that, in the absence of MAP3K2, upon blocking methylation at K260, and with inhibition of ERK1/2 activation, SMYD3 levels are stabilized through inhibition of the proteasome. This finding indicates that SMYD3 abundance can be controlled posttranslationally and that SMYD3 levels are maintained downstream of MAP3K2 through a proteasome-dependent pathway. There is some evidence that SMYD3 can interact with or may be regulated by ubiquitin ligase machinery (*74*); however, the significance of these factors in regulating SMYD3 protein abundance has not been determined. Nonetheless, our findings portend that other Ras-dependent cancers with increased abundance of SMYD3 are also likely to maintain a feedback loop driving high SMYD levels dependent on persistent activation of MAP3K2/MEK/ERK signaling.

There is accumulating evidence that aberrant activation of Ras/MAPK signaling is a common signature of advanced-stage PCa (*2*, *5*, *6*), likely through diverse mechanisms that result in gene amplification, altered gene expression, or disrupted protein abundance of pathway components and regulators. These observations support recent investigations of targeting MAPK signaling particularly for treating mCRPC with pathway blockade (*75*), such as with the MEK1/2 inhibitor trametinib, which has previously been approved for clinical use in melanoma and is currently in a phase 2 clinical trial for patients with CRPC (*24*). Our data have extended the characterization of SMYD3 as a MAPK regulator to another cancer type and link its catalytic activity to aggressive, EMT-associated phenotypes of PCa. This implies that pharmacological inhibition of SMYD3 activity provides an additional mechanism for disrupting the Ras/MAPK signaling axis that may be critical in metastatic disease, particularly CRPC. Moreover, in mouse models of Ras-induced lung adenocarcinoma, loss of SMYD3 augments the effectiveness of trametinib in blocking tumorigenesis (*18*), highlighting the possibility of combined therapies that may evade resistance. Together, our findings reveal a critical role for the SMYD3-MAP3K2 signaling axis in driving disease progression and aggressiveness in PCa and support continued investigation into the role of SMYD3 in additional models of PCa and its potential as a therapeutic target for metastatic disease.

## MATERIALS AND METHODS

### Cell culture, transfections, and viral transductions

Androgen-dependent and refractory PCa cell lines LNCaP and PC-3 were grown in RPMI medium (Corning) supplemented with 10% fetal bovine serum (FBS) (GIBCO) and penicillin/streptomycin (100 U/ml). Human embryonic kidney (HEK) 293T and NIH3T3 cells were cultured in Dulbecco’s modified Eagle’s medium (Corning) with 10% FBS (GIBCO) and penicillin/streptomycin (100 U/ml). All cells were cultured at 37°C in a humidified incubator with 5% carbon dioxide (CO_2_). For transient expression, cells were transfected using Attractene transfection reagent (Qiagen) and collected after 24 to 36 hours. To generate stable cell lines for shRNA knockdown (with pLKO-TetOn and pSicoR) and inducible reconstitution (with pInducer20), lentiviral particles were produced by cotransfection of HEK293T cells with the plasmids and packaging vectors pVSVg and pΔ8.2. After 48 hours, virus was collected, filtered, and used for transduction of target cells along with polybrene (8 μg/ml). Infected cells were selected by treatment with either puromycin (2 μg/ml) or neomycin (200 μg/ml) for 7 days.

### Plasmids

For plasmids used in PCa cells, coding sequences of full-length *SMYD3*, *MAP3K2*, and *VIMENTIN* were cloned into pInducer20 vector through Gateway cloning (Invitrogen). Wild-type coding sequences were amplified from cDNA, and SMYD3 F183A and MAP3K2 K260A point mutants were generated using site directed mutagenesis. shRNA sequences targeting the 3′UTR of SMYD3 were cloned into pLKO-TetOn and pSicoR plasmids, and the shRNA targeting MAP3K2 was cloned into pLKO-TetOn plasmid. Oligo sequences used for shRNA-mediated knockdown are listed in table S1.

### Cell viability assay

CellTiter-Blue (Promega) was used to monitor cell viability following the manufacturer’s recommendations. Where indicated, the SMYD3 inhibitor EPZ031686 (MedChem Express) or the catalytic ERK1/2 inhibitor BVD-523 (MedChem Express) were used.

### Migration assay

Collective cell migration assays measured the time required by cells to close a cell-free 500-μm gap. Cells were seeded at 6 × 10^5^ cells/ml on four-well insert 35-mm plates (ibidi) and incubated for 24 hours until reaching 90% confluency. The insert separating the adherent cells was removed, and live imaging was performed using a BioTek BioSpa Cytation 5 Live Cell Analysis Imager (Agilent). Images were taken every 30 min until the gap closed. Migration rates were determined using ImageJ as described previously in square micrometers per hour (*76*). To assess migration independently from cell proliferation, aphidicolin (3 μg/ml) was added for 36 hours before starting the assay, and flow cytometry was used to monitor cell cycle stage using a BD FACSMelody Cell Sorter, as previously described (*77*, *78*).

### Adhesion assay

For studying cell-fibronectin adhesion, 96-well plates were coated with human fibronectin (5 μg/ml). A total of 5000 cells per well were seeded and allowed to adhere for 60 to 90 min at 37°C in a humidified incubator with 5% CO_2_. Unadhered cells were washed away with phosphate-buffered saline (PBS), adherent cells were stained with crystal violet, and imaged using a BioTek Cytation 5 Cell Imaging Multimode Reader (Agilent).

For cell-cell adhesion assays, NIH3T3 or PCa cells were grown to 100% confluency to coat the well in 96-well plates. PCa cells (−/+ doxycycline or drug treatment) were permanently fluorescently labeled by incubating with 10 μM calcein-acetoxymethyl ester (AM) (Invitrogen) for 60 to 90 min and seeded at 5000 cells per well on top of the confluent layer of unlabeled cells. Cells were allowed to adhere for 3 hours at 37°C in a humidified incubator with 5% CO_2_. Unadhered cells were washed away with PBS, and adherent cells were imaged using a BioTek Cytation 5 Cell Imaging Multimode Reader (Agilent).

### Invasion assay

Invasion assays were performed using Transwell inserts (8-μm pore, Corning) coated with a 1:3 Matrigel (Corning) dilution in media. PCa cells were seeded at 1 × 10^6^ cells/ml in the top chamber and allowed to invade through the membrane for 24 hours toward the bottom chamber containing 20% FBS medium as chemoattractant. After 24 hours, invaded cells were fixed with 4% paraformaldehyde (PFA) for 15 min and stained with crystal violet. Cells were imaged using a BioTek Cytation 5 Cell Imaging Multimode Reader (Agilent).

### Soft agar assay

Assessment of anchorage-independent growth was performed by suspending 5000 cells/ml in 0.35% agarose in media and adding them to 0.5% agar in 96-well plates. For inducible expression, doxycycline was added to the feeding media. Cells were incubated for 2 weeks and then imaged using a BioTek Cytation 5 Cell Imaging Multimode Reader (Agilent).

### Spheroid formation

Three-dimensional tumor spheroids of PC-3 and LNCaP cells were generated by hanging drop method or scaffold-based method as previously described (*79*). Briefly, for the hanging drop method, 5000 cells were deposited as 10-μl drops on nonadherent sterile lids with PBS in the bottom of the dish. Spheroidal growth was monitored for 1 week using light microscopy.

### Xenografts

NSG (NOD.Cg*-Prkdc^scid^ Il2rg^tm1Wjl^/SzJ*) mice were acquired from the Jackson Laboratory (strain no.: 005557). Mice were housed in the University of Maryland Baltimore County (UMBC) animal care facility to acclimatize for a week before being enrolled in respective xenograft studies. Fourteen NSG mice were enrolled in flank xenograft studies, and 42 NSG mice were enrolled in two separate orthotopic xenograft studies. All subcutaneous and orthotopic implantation procedures were performed according to the Institutional Animal Care and Use Committee at UMBC (protocol CB793).

For flank xenografts, 5 × 10^6^ PCa cells (100 μl) in Matrigel (1:1 ratio; Corning) were subcutaneously injected into the hind flank of immunocompromised NSG mice. SMYD3 repression was maintained by providing water with doxycycline (200 μg/ml) throughout the experiment. Tumor size was measured every other day using a digital caliper, and respective tumor volume was estimated using the ellipsoid formula (length × width^2^).

For orthotopic xenografts, 500,000 cells (30 μl) in Matrigel (1:3 ratio; Corning) were surgically injected into the anterior prostate lobe of anesthetized NSG mice. Cohorts requiring doxycycline treatment were provided water with doxycycline (200 μg/ml) starting 7 days after surgery. Tumor volume was digitally monitored throughout the study until mice met criteria for euthanasia.

### Cell extracts, immunoprecipitation, and immunoblotting

For whole-cell extracts, cells were lysed in radioimmunoprecipitation assay (RIPA) buffer [50 mM tris-HCl (pH 8), 150 mM NaCl, 1 mM EDTA, 1% NP-40, 0.1% SDS, and 0.5% sodium deoxycholate] with 1 mM phenylmethylsulfonyl fluoride and protease and phosphatase inhibitor cocktails (Thermo Fisher Scientific). Total protein concentration was determined by bicinchoninic acid (BCA) assay (Pierce). Subcellular fractionation and immunoprecipitation (IP), experiments were performed as previously described (*18*). IP extracts were incubated with specific antibody-bound protein A/G magnetic beads (Pierce) overnight at 4°C and then eluted with SDS loading buffer.

Protein extraction from fresh tissue was performed as previously described (*80*). Briefly, 20-mm^3^ tissue was cross-sectionally cut with sterile scalpel and gently washed with ice-cold 70% ethanol twice and ice-cold water once. For whole-tissue extract, tissue was then lysed with lysis buffer [8 M urea, 100 mM tris-HCl (pH 7.5), and 100 mM dithiothreitol] using a tissue homogenizer and sonicated with three bursts of 10 s each with intermittent cooling on ice. For tumor IPs, extracts were prepared by homogenizing and sonicating (three bursts of 30 s each) tissue sections in RIPA buffer. Samples were centrifuged at 16,000*g* for 15 min, and supernatant as total protein was collected. Total protein concentration was determined by BCA assay (Pierce). IP lysates were precleared by incubating with protein A/G magnetic beads (Pierce) for 30 min at 4°C and then incubated with specific antibody-bound magnetic beads overnight at 4°C and eluted with SDS loading buffer. Proteins were resolved by SDS–polyacrylamide gel electrophoresis, transferred to polyvinylidene fluoride membrane for immunoblotting, and imaged using a LiCor Odyssey imaging system. All antibodies used for immunoblotting are listed in table S2.

### Immunofluorescence microscopy

Cells were seeded on poly-l-lysine–coated coverslips (Corning), allowed to adhere for 24 hours, washed with PBS, and fixed with 4% PFA for 15 min at room temperature. After washing three times with PBS, the fixed cells were blocked with 5% normal goat serum and 0.3% Triton X-100 in PBS for 30 min at 37°C. Coverslips were transferred to a humidified chamber and incubated with primary antibody overnight at 4°C. After three washes with IF wash buffer [10 mM tris (pH 7.4), 150 mM NaCl, and 0.05% Tween 20], cells were incubated with secondary antibody for 1 hour at 37°C, washed three times with wash buffer, and incubated with phalloidin (Biotium) for 45 min at room temperature. Last, cells were washed three times with PBS and mounted on a glass slide with 4′,6-diamidino-2-phenylindole (Vectashield). Cells were visualized with a Zeiss LSM 900 confocal microscope, and image analysis was performed with ImageJ.

### Reverse transcription quantitative PCR

Total RNA was isolated using the Lucigen RNA Isolation Kit according to the manufacturer’s instructions, and 1 μg of RNA was used to generate cDNA using the qMax cDNA synthesis kit (Accuris). Quantitative PCR (qPCR) was performed as previously described (*81*, *82*). Gene specific primers (20 μM) were added to 0.5 μl in 1× qMax Green Low ROX qPCR mix (Accuris) in 10-μl reactions and amplified using a Bio-Rad CFX384 instrument. Data are representative of a minimum of three biological samples with three technical replicates per sample. Relative gene expression was normalized to *GAPDH*. Oligo sequences for qPCR are listed in table S3.

### Bioinformatic analysis

Gene expression profiles from TCGA–Prostate Adenocarcinoma and GTEx projects were obtained from The University of California, Santa Cruz Xena genome browser (*83*). Gene expression data for the following PCa datasets were obtained from National Center for Biotechnology Information Gene Expression Omnibus: Gulzar *et al.* (GSE40272) (*30*), Ross-Adams *et al.* (GSE70768) (*31*), Taylor *et al.* (GSE21032) (*2*), Grasso *et al.* (GSE21032) (*84*), and Roudier *et al.* (GSE21032) (*85*). Statistical analysis was performed on the mRNA expression profiles.

### Statistical analyses

All experiments were performed with minimum three independent biological replicates and are presented as means ± SD in column graphs with individual data points. Survival studies were analyzed by Kaplan-Meier curves and log-rank (Mantel-Cox) tests. One-way analysis of variance (ANOVA) with post hoc test or two-tailed Student’s *t* test was used to compare means of two or more samples using Prism 8.0.2 software (GraphPad Software). For all analyses, results were considered statistically significant with *P < 0.05, **P < 0.01, and ***P < 0.001.
