# Supplementary material for: The SMYD3-MAP3K2 signaling axis promotes tumor aggressiveness and metastasis in prostate cancer
Source: Sci Adv. 2023 Nov 17;9(46):eadi5921. doi: 10.1126/sciadv.adi5921 (PMC10656069; doi:10.1126/sciadv.adi5921)
Supplement: Supplementary file 1 — Figs. S1 to S5 Tables S1 to S3 [file sciadv.adi5921_sm.pdf]

Science Advances
AAAS

Supplementary Materials for

The SMYD3-MAP3K2 signaling axis promotes tumor aggressiveness and metastasis in prostate cancer

Sabeen Ikram *et al.*

Corresponding author: Erin M. Green, egreen@umbc.edu

Sci. Adv. **9**, eadi5921 (2023)
DOI: 10.1126/sciadv.adi5921

This PDF file includes:

Figs. S1 to S5
Tables S1 to S3

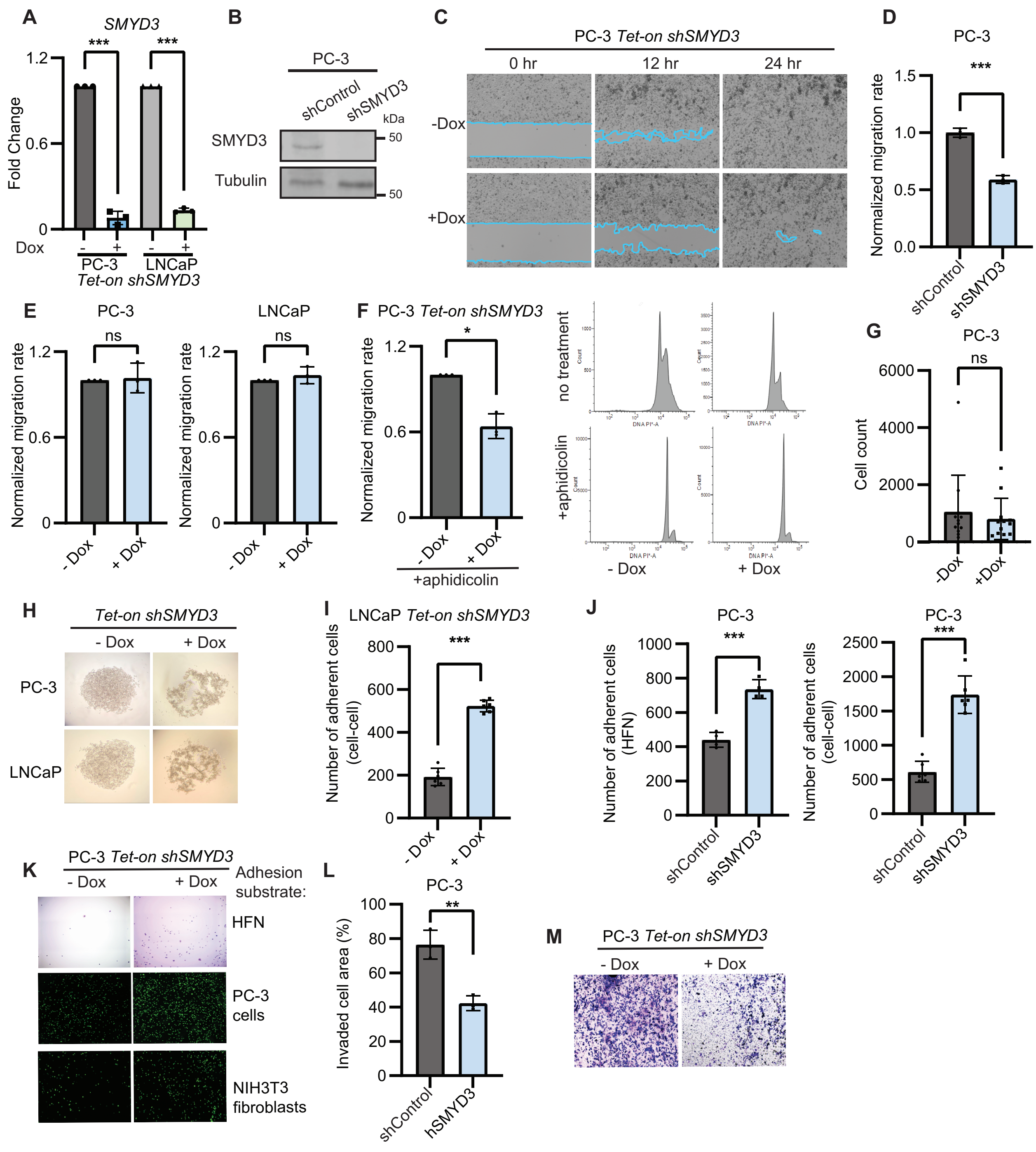

Fig. S1: Loss of SMYD3 inhibits prostate cancer progression in multiple cell lines.

A Fold change of *SMYD3* mRNA abundance in PC-3 and LNCaP *Tet-on shSMYD3* cells -/+ dox treatment measured by RT-qPCR. Significance was evaluated using one-way ANOVA and Tukey's multiple comparisons test. **B** Immunoblot of SMYD3 levels from PC-3 *shControl* (empty vector) and *shSMYD3* cells. **C** Representative images of PC-3 *Tet-on shSMYD3* cells migrating at

12 and 24 hours. **D** Normalized migration rate of PC-3 *shControl* and *shSMYD3* cells (n=3). **E** Normalized migration rate of PC-3 and LNCaP cells -/+ dox treatment (n=3). **F** Normalized migration rate of aphidicolin treated PC-3 *Tet-on shSMYD3* cells -/+ dox treatment (n=3) and flow cytometry profiles of DNA content in cells -/+ aphidicolin treatment. **G** Soft-agar assay quantifying anchorage-independent growth of PC-3 cells -/+ dox treatment (n=12). **H** Representative images of spheroids of PC-3 and LNCaP *Tet-on shSMYD3* cells at 48 hours -/+ dox treatment. **I** Adhesion of LNCaP *Tet-on shSMYD3* cells -/+ dox treatment to wildtype LNCaP cells (n=6). **J** Adhesion of PC-3 *shControl* and *shSMYD3* cells to HFN (n=4) (*left*) and to wildtype PC-3 cells (n=3) (*right*). **K** Representative images of crystal violet or calcein AM stain of PC-3 *Tet-on shSMYD3* cells -/+ dox treatment adhered to HFN, wildtype PC-3 cells, or to NIH3T3 fibroblasts. **L** Invasion capacity of PC-3 *shControl* and *shSMYD3* cells using transwell invasion assay (n=3). **M** Representative images of crystal violet staining of transwell invasion assay of PC-3 *Tet-on shSMYD3* cells -/+ dox treatment. For migration, soft agar, and adhesion assays, significance was evaluated using two-tailed unpaired student's t-test. For all panels, error bars represent standard deviation (SD) and *p*-values are indicated as follows: *< 0.05, **< 0.01, ***<0.001.

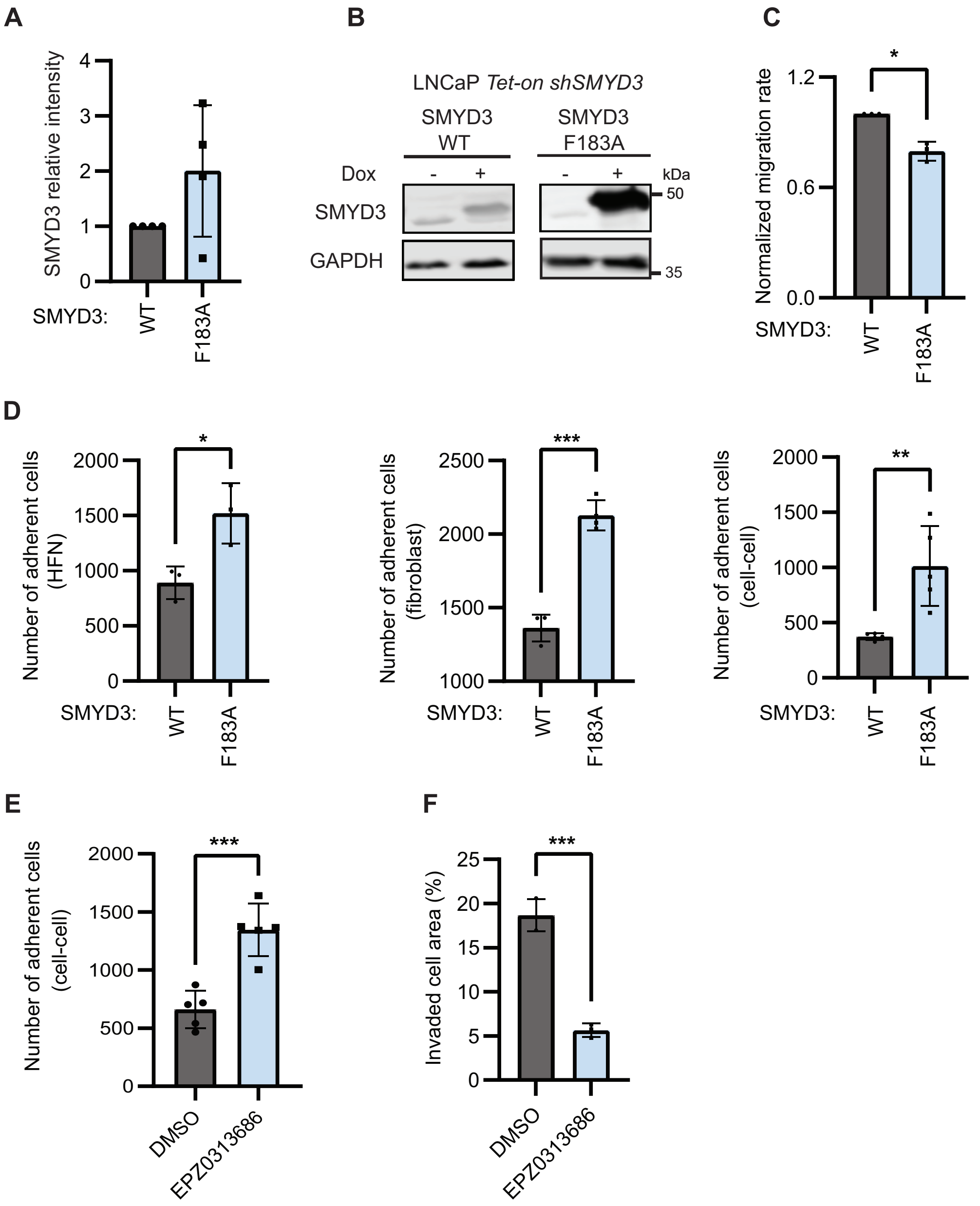

Fig. S2: Inhibiting SMYD3 catalytic activity attenuates tumorigenic properties of LNCaP cells.

A Intensity of SMYD3 relative to GAPDH and normalized to SMYD3$_{WT}$ abundance in PC-3 cells. **B** SMYD3 abundance shown by immunoblot in LNCaP *Tet-on shSMYD3* cells reconstituted with either wildtype SMYD3 (*Tet-on SMYD3$_{WT}$)* or SMYD3 with catalytic mutation F183A (*Tet-on SMYD3$_{F183A}$*). **C** Normalized migration rate of LNCaP *Tet-on shSMYD3* reconstituted with SMYD3$_{WT}$ and SMYD3$_{F183A}$ treated with dox (n=3). **D** Adhesion of LNCaP *Tet-on shSMYD3* cells treated with dox and reconstituted with SMYD3$_{WT}$ and SMYD3$_{F183A}$ to HFN (n=3) (*left*), to wildtype LNCaP cells (n=5) (*middle*), and to NIH 3T3 fibroblasts (n=3) (*right*). **E** Adhesion of LNCaP cells treated with DMSO or SMYD3 inhibitor (SMYD3i; 500 nM EPZ0313686) to wildtype LNCaP (n=5). **F** Invasion capacity of LNCaP cells treated with DMSO or SMYD3i (n=3). For all panels, error bars represent standard deviation (SD), significance was evaluated

using two-tailed unpaired student's t-test, and *p*-values are indicated as follows: $*< 0.05$, $**< 0.01$, $***<0.001$.

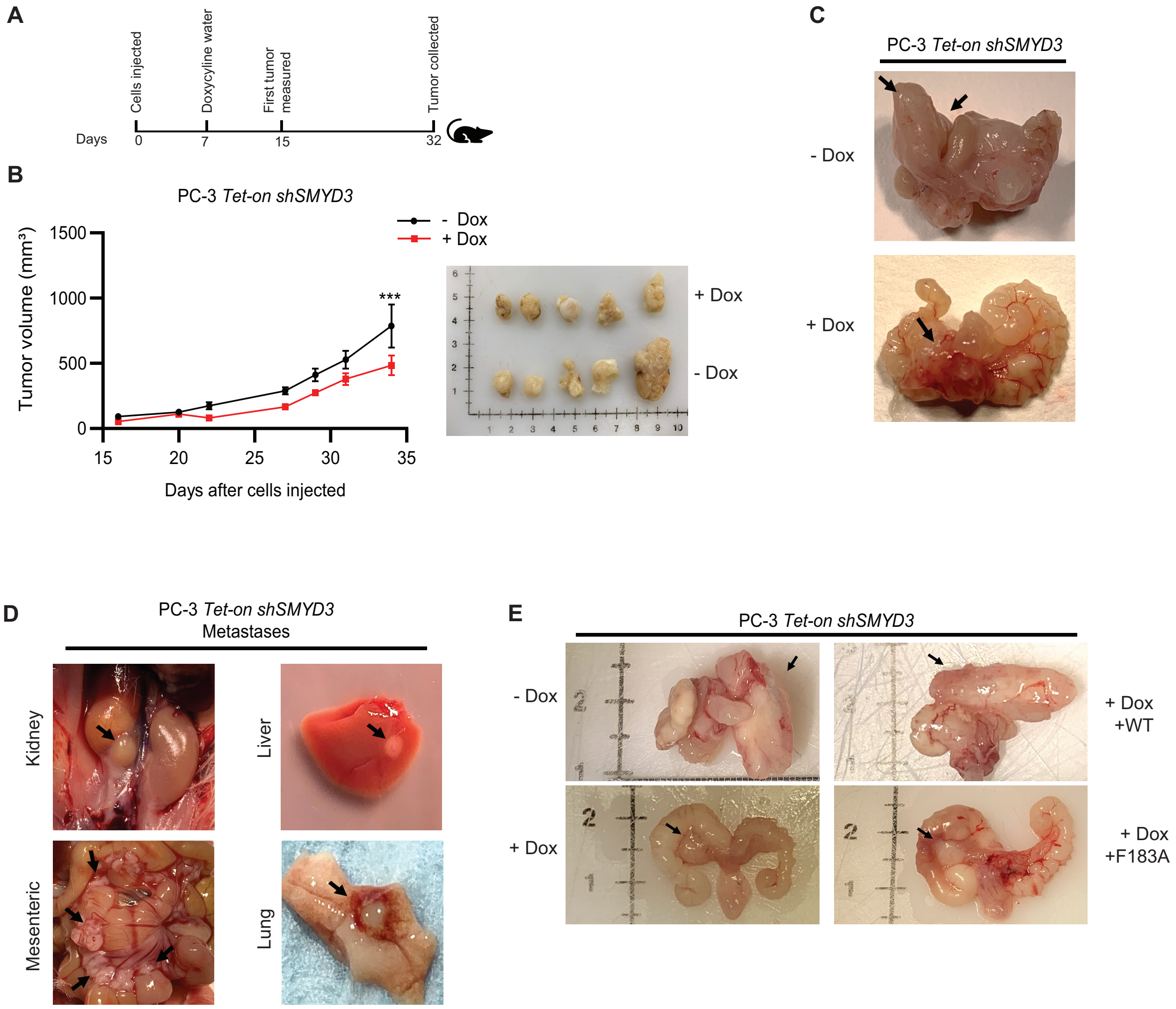

Fig. S3: SMYD3 promotes tumor growth and metastases in prostate cancer xenograft mouse models.

A Protocol for PC-3 *Tet-on shSMYD3* flank xenografts in NSG mice -/+ 200 µg/mL dox treatment in water (n=8 and n=6, respectively). **B** Progression of flank tumor volume (*left*), and representative images of tumors collected (*right*). Error bars represent SD, significance was evaluated using two-way ANOVA and Sidak's multiple comparisons test. *p*-values are indicated as follows: *< 0.05, **< 0.01, ***<0.001. **C** Representative images of primary prostate tumors with urogenital sinus (UGS) collected from necropsied mice with PC-3 *Tet-on shSMYD3* orthotopic xenograft implants -/+ dox water treatment. Black arrowheads indicate primary tumors. **D** Representative images of metastases in liver, lung, mesentery, and kidney from orthotopic implants of PC-3 *Tet-on shSMYD3*. Black arrowheads indicate site of metastases. **E** Representative images of primary prostate tumor with UGS from orthotopic xenograft implants of PC-3 *Tet-on shSMYD3* and reconstituted *Tet-on* $SMYD3_{WT}$ and $SMYD3_{F183A}$ cells. Black arrowheads indicate primary tumors.

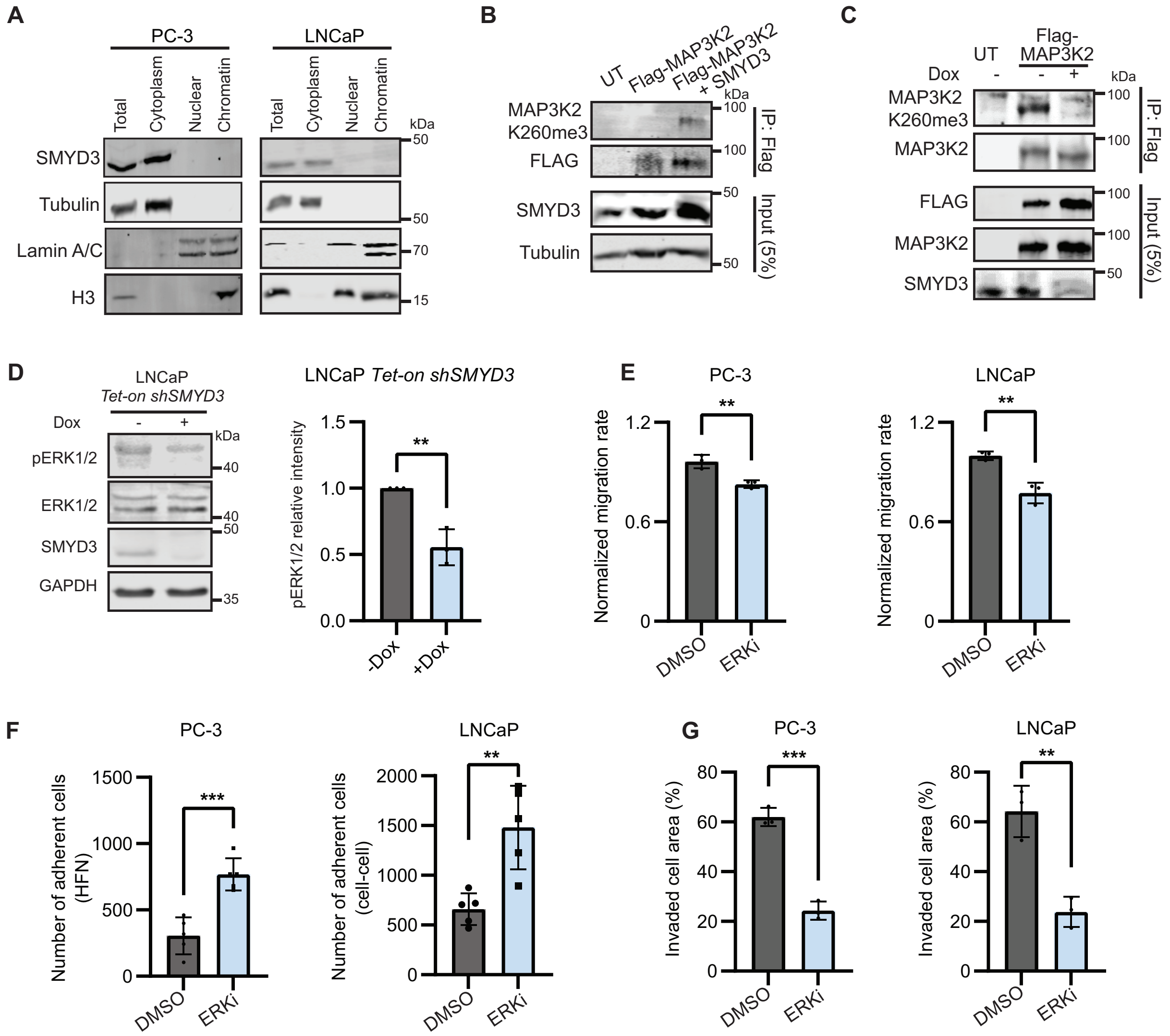

Fig. S4: SMYD3 methylates MAP3K2 in the cytoplasm and disruption of MAPK signaling via ERK inhibition decreases tumorigenic properties of PCa cells.

A Immunoblots showing biochemical fractionation of PC-3 (*left*) and LNCaP (*right*) cells into cytoplasmic, nuclear and chromatin fractions probed with anti-SMYD3, anti-tubulin, anti-lamin A/C and anti-H3 antibodies. **B** Immunoprecipitation (IP) of Flag-MAP3K2 from PC-3 cells overexpressing Flag-MAP3K2 and SMYD3 compared to an untransfected (UT) control. IP immunoblots probed with anti-MAP3K2 K260me3 and anti-Flag antibodies. **C** IP of Flag-MAP3K2 from PC-3 *Tet-on shSMYD3* cells -/+ dox treatment compared to an untransfected (UT) control. IP immunoblots probed with anti-MAP3K2 K260me3, anti-Flag and anti-MAP3K2 antibodies. **D** Immunoblots using anti-phosphoERK1/2, anti-ERK1/2, and anti-SMYD3 in LNCaP *Tet-on shSMYD3* cells -/+ dox treatment. Intensity of phosphoERK1/2 signal relative to total ERK1/2 normalized to GAPDH plotted on the right. **E** Normalized migration rate of DMSO and ERK inhibitor (ERKi; 4µM BVD-523) treated PC-3 and LNCaP cells (n=3). **F** Adhesion of DMSO and ERKi (BVD-523) treated PC-3 cells to HFN (n=5), and LNCaP to wildtype LNCaP cells (n=5). **G** Invasion capacity of DMSO and ERKi (BVD-523) treated PC-3 and LNCaP cells (n=3). For all panels, error bars represent SD, significance was evaluated using two-tailed unpaired student's t-test, and *p*-values are indicated as follows: *< 0.05, **< 0.01, ***<0.001.

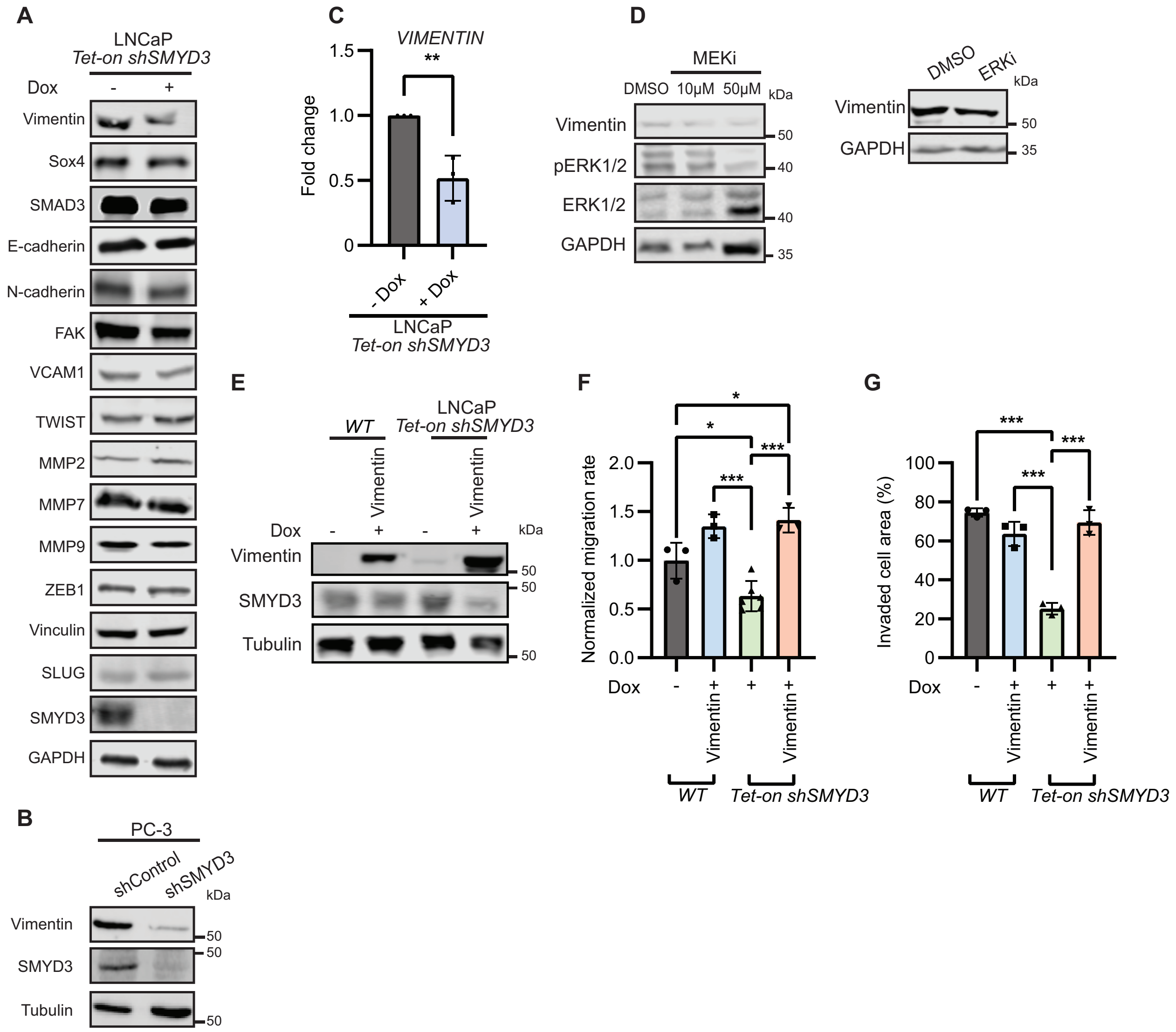

Fig. S5: SMYD3 regulates the abundance of vimentin via MAPK signaling.
A Immunoblots of EMT-associated proteins from LNCaP *Tet-on shSMYD3* cells -/+ dox treatment. **B** Immunoblot of vimentin levels from PC-3 *shControl* (empty vector) and *shSMYD3* cells. **C** Fold change of *vimentin* mRNA abundance in LNCaP *Tet-on shSMYD3* cells -/+ dox treatment measured by RT-qPCR. **D** Immunoblots of vimentin and pERK1/2 from LNCaP cells treated with 10μM and 50μM MEKi (trametinib) (*left*), and from LNCaP cells treated with 4μM ERKi (BVD-523) (*right*). **E** Immunoblotting of vimentin levels in LNCaP *WT* and LNCaP *Tet-on shSMYD3* cells each with *Tet-on VIMENTIN* -/+ dox treatment. **F** Normalized migration rate of LNCaP *WT* (n = 3) and LNCaP *Tet-on shSMYD3* (n = 6) cells each with *Tet-on VIMENTIN* (n = 3 for each cell type) -/+ dox treatment. **G** Invasion capacity of LNCaP *WT* and LNCaP *Tet-on shSMYD3* (cells each with *Tet-on VIMENTIN* (n = 3 for each cell type) -/+ dox treatment. For all panels, error bars represent standard deviation (SD), significance was evaluated using an unpaired student's t test (**B**) or one-way ANOVA and Tukey's multiple comparisons test (**F, G**), and *p*-values are indicated as follows: *< 0.05, **< 0.01, ***<0.001.

Table S1: shRNA sequences

Gene	Target sequence
SMYD3 (pLKO Tet-On)	TGAACGCAGTCAGAGGGAAATTCAAGAGATTTCCCTCTGACTGC GTTCTTTTTC
SMYD3 (pSicoR)	TGCGTGTGTCTTTGTTGAATTTCAAGAGAATTCAACA AAGACACACGCTTTTTC
MAP3K2	GGATGATTTCACTAGGCATCTCGAGATGCCTAGTGAAATCATCC

Table S2: Antibodies

Antibody	Source	Identifier
B-actin	Sigma-Aldrich	A5316
E-cadherin	Proteintech	20874-1-AP
ERK1/2	Cell Signaling	9102
FAK	Proteintech	12636-1-AP
GAPDH	Proteintech	60004-1-Ig
Lamin A/C	Cell Signaling	4777
MAP3K2	Invitrogen	SC68-02
MAP3K2me2/3	(*18*)	N/A
MMP2	Abclonal	A19080
MMP7	Abclonal	A0695
MMP9	Abclonal	A0289
N-cadherin	Proteintech	22018-1-AP
pERK1/2	Cell Signaling	9101
Phalloidin	Biotium	00045
SLUG	Abclonal	A1057
SMAD3	Abclonal	A19115
SMYD3	Abcam	EPR11107(2)
SNAI1	Proteintech	13099-1-AP
SOX4	Abclonal	A21222
Tubulin	Proteintech	66031-1-Ig
TWIST1	Proteintech	25465-1-AP
Ubiquitin	RnD Systems	A-104
V-CAM1	Proteintech	11444-1-AP
Vimentin	Thermofisher Scientific	MA5-11883
Vinculin	Proteintech	66305-1-Ig
Zeb1	Abclonal	A1500

Table S3: qRT-PCR primer sequences

Gene	Primer sequence
SMYD3	5’ TTCCCGATATCAACATCTACCAG 3’
	5’ AGTGTGTGACCTCAATAAGGCAT 3’
GAPDH	5’ GTCTCCTCTGACTTCAACAGCG 3’
	5’ ACCACCCTGTTGCTGTAGCCAA 3’
Vimentin	5’ AGGCAAAGCAGGAGTCCACTGA 3’
	5’ ATCTGGCGTTCCAGGGACTCAT 3’
